# Different Isoforms of PML-RARA Chimeric Protein in Patients with Acute Promyelocytic Leukemia: Survival Analysis per Demographic Characteristics, Clinicohematological Parameters, and Cytogenetic Findings

**DOI:** 10.30699/IJP.2023.2007229.3145

**Published:** 2023-12-15

**Authors:** Sarah Siahbani, Akbar Safaei, Masoumeh Faghih, Marzieh Hosseini, Afsaneh Fendereski, Behnaz Valibeigi, Ahmad Monabati

**Affiliations:** 1 *Molecular Pathology and Cytogenetic Ward, Department of Pathology, School of Medicine, Shiraz University of Medical Sciences, Shiraz, Iran*; 2 *Department of Biostatistics, School of Health, Health Sciences Research Center, Mazandaran University of Medical Sciences, Sari, Iran*

**Keywords:** APL, AML-M3, bcr1, bcr3, PML-RARA isoforms, Iran, Survival analysis

## Abstract

**Background & Objective:**

Acute Promyelocytic Leukemia (APL) is a medical emergency with potentially fatal complications. APL primarily results from a chromosomal translocation (t(15;17)(q22;q21)), leading to the formation of the PML-RARA fusion gene with three possible isoforms. This study aims to investigate the characteristics of Iranian APL patients, the distribution of PML-RARA isoforms, and survival analysis.

**Methods:**

We included 145 consecutive eligible patients in this study. Data were collected through archived documents and phone inquiries, following consent. Subsequently, we analyzed the data using SPSS software version 26.0.

**Results:**

We examined 75 men and 70 women, with a mean age of 34 years (range: 2-78 years). Besides t(15;17) (q22;q21), 45.6% had other chromosomal abnormalities. The prevalence of bcr1 and bcr3 isoforms was 73% and 27%, respectively. bcr3 correlated with higher white blood cell (WBC) counts, additional chromosomal abnormalities, and faster Complete Hematologic Response (CHR). Early death occurred in approximately 36% of all patients. The mean overall survival time was 73.5 months, with 120-month survival rates of 53.8% for all patients and 83.9% for those who achieved CHR. Univariate analysis identified old age, relapse, lower platelet (PLT) counts, higher WBC counts, and leukocytosis as survival risk factors. However, in multivariate analysis, only old age and higher WBC counts were identified as adverse prognostic factors.

**Conclusion:**

In Iranian APL patients, bcr1 predominates, while bcr3 correlates with higher WBC counts, high-risk categorization, additional chromosomal abnormalities, and faster CHR. Survival is negatively impacted by old age, relapse, lower PLT counts, higher WBC counts, and leukocytosis.

## Introduction

Acute promyelocytic leukemia (APL) is a distinct subtype of acute myeloid leukemia (AML), classified as AML-M3 in the French-American-British (FAB) classification. It accounts for approximately 5-15% of all patients with AML. It is morphologically characterized by abnormal promyelocytes (1-5). APL is a hemato-oncologic emergency, which requires rapid initiation of treatment due to potentially fatal complications, including disseminated intravascular coagulation (DIC) (6, 7). Therefore, in cases of clinical suspicion, treatment should be initiated even before cytogenetic/molecular diagnostic confirmation (8).

The majority of APL patients reported so far (about 99%) are characterized by a balanced reciprocal translocation between chromosomes 15 and 17, resulting in the PML-RARA fusion gene (1, 9, 10). In a small number of APL patients (about 1%), the RARA gene (located on chromosome 17) is combined with another partner gene, typically involved in regulating cell growth and/or cell differentiation processes. Currently, at least fifteen partner genes for RARA have been identified, with the ZBTB16 gene being the most commonly reported (11, 12).

The PML gene, located on chromosome 15, can experience breaks in one of the following breakpoint cluster regions (bcrs):

1. bcr1 (intron 3; Long (L) isoform)
2. bcr2 (exon 6; Variable (V) isoform)
3. bcr3 (intron 6; Short (S) isoform)

Molecular studies have revealed the existence of three different possible isoforms for the PML-RARA gene, corresponding to these breakpoints: bcr1, bcr2, and bcr3 (1).

 Multiple studies conducted in different countries around the world have reported variable frequencies for three PML-RARA isoforms, with the predominance of the bcr1 isoform, accounting for approximately 50-55% of cases, followed by the bcr3 isoform at 27-49%, and the bcr2 isoform at 8-20% (13-19). However, two published reports from India have indicated the predominant isoform to be S (bcr3), and these differences have been attributed to genetic factors as well as environmental factors unique to different societies (20, 21).

Transcription of the PML-RARA gene leads to the expression of a chimeric oncoprotein called PML-RARα, which is involved in the pathogenesis of APL disease (22, 23). Along with the transcription of the entire PML-RARA gene, the transcription of its PML portion also takes place, resulting in the translation of an abnormal and shorter PML protein with impaired function (23-26). Normally, domains present in RARα allow this protein to heterodimerize with the retinoid X receptor α (RXRα) protein and bind to DNA sites called retinoic acid response elements (RARE). After translocation, RARα domains can still bind to RARE and subsequently bind to the RXRα protein, exerting a dominant negative effect on normal RARα/RXRα transcriptional activity (11, 23). Furthermore, these newly formed RARα proteins can homodimerize, providing them with the capability to regulate a wide range of genes not typically affected by normal RARα. Additionally, PML-RARα chimeric proteins act as potent repressors of transcription influenced by retinoic acid signaling and interfere with myeloid cell differentiation, leading to an arrest at the promyelocyte stage (6, 23).

Multicenter studies conducted from the 1990s to the present have consistently demonstrated the effectiveness of All-Trans Retinoic Acid (ATRA) in combination with chemotherapy agents, as well as ATRA plus Arsenic Trioxide (ATO), either alone or in conjunction with chemotherapy, in the treatment of APL (7, 27). ATRA and ATO bind to specific sites on PML-RARα or abnormal PML oncoproteins (16) and induce their degradation through different mechanisms (28).

Although the clinical consequences, effects on therapeutic responses, and prognosis resulting from different PML-RARA isoforms are not yet definitively defined (23, 29), scientific literature suggests the importance of identifying PML-RARA isoforms during disease diagnosis. In this regard, several previous studies have reported that the S isoform is associated with a shorter remission duration (18, 30-35) and overall survival(18, 30-34, 36) when compared to the L isoform. Additionally, studies have revealed associations between the S isoform and a younger age of disease onset(37), higher WBC count (17, 18, 30, 35, 38), the hypogranular variant (M3v)(17, 18, 30), CD34 expression(39), FLT3 gene mutation (30, 40), resistance to differentiating agents (i.e., ATRA and ATO)(41), and a higher risk of relapse(33, 36), especially extramedullary relapse (42). Conversely, numerous studies have found no significant differences between bcr isoforms with regard to the presence of these associations (1, 9, 13, 20, 21, 43, 44). 

Due to the limited number of available studies in the Middle East region, including Iran, as well as the small sample size of APL patients in these few studies, the demographic characteristics, clinical manifestations, distribution of bcr isoforms, and prognostic factors influencing overall survival rates remain unclear. Consequently, this study was conducted to ascertain the distribution of PML-RARA isoforms and explore potential relationships with other studied parameters. Additionally, survival analysis was performed in a retrospective cohort of APL patients in the southern region of Iran.

## Material and Methods

This original article presents the findings of a retrospective single-center observational and analytical study that encompassed all eligible patients admitted to the Department of Molecular Pathology and Cytogenetics at Shiraz University of Medical Sciences, situated in southern Iran, over ten years spanning from February 2012 to June 2022.

The research plan received approval from the Ethics Committee of the Research and Technology Center of Shiraz University of Medical Sciences (Approval ID: IR.SUMS.MED.REC.1401.157). All procedures strictly adhered to ethical guidelines, with a primary focus on maintaining patient confidentiality. Required data was extracted from archived patient records, and additional information was collected through telephone interviews with patients or their families (in cases of deceased patients). Prior to data collection, the study's objectives were clearly explained, and participants' voluntary consent was obtained.

It's important to note that in line with international standard guidelines for induction, consolidation, and maintenance treatment protocols (29, 45-50), all necessary medical services were diligently provided to each patient.

Inclusion criteria: This study included all consecutive, newly diagnosed de novo APL patients who were confirmed to have PML-RARA mRNA presence through PCR testing. The inclusion period extended from February 2012 to June 2022.

Exclusion criteria: The following groups of patients were excluded from the study:

• APL patients with a history of another type of leukemia.

• Treatment-related APL patients.

• Relapsed cases, where the primary disease onset occurred before the desired time (February 2012 to June 2022).

**Demographic Information**

All relevant demographic and clinical information, including age, gender, family history of malignancies, and onset symptoms (ecchymosis/petechia, bleeding, and thromboembolic events like Cerebrovascular accident (CVA), Deep vein thrombosis (DVT), and Pulmonary thromboembolism (PTE), as well as other non-specific symptoms (such as fever, infection, pallor, weakness, fatigue, bone pain, lethargy, anorexia, weight loss, etc.)), were meticulously collected. Additionally, the time taken to achieve the initial complete hematologic response (CHR) was recorded in accordance with established protocols (51, 52).

**Whole Blood Analysis and Risk Classification**

Upon the patients' initial hospitalization, a complete blood count with differential (CBC Diff) was performed. Patients were classified into three risk groups based on the recurrence risk classification proposed by Sanz* et al. *in 2000(7) :

• High risk: WBC > 10,000/µL

• Intermediate/Standard risk: WBC < 10,000/µL and PLT < 40,000/µL

• Low/Standard risk: WBC < 10,000/µL and PLT > 40,000/µL

**Histomorphologic Evaluation**

To examine the histomorphology of bone marrow and peripheral blood cells, the corresponding slides were stained using Wright's method.

**Flow Cytometry**

Immunophenotyping of heparinized bone marrow aspirate cells was performed with a flow cytometry device (BD FACSCalibur™ System; BD Biosciences, USA) using a standard four-color method. Cell Quest Pro software was used for data analysis.

**Cytogenetic and Molecular Tests**

All cytogenetic(53, 54) and molecular(55-58) tests were conducted in accordance with established routine methods, which were documented as standard operating procedures (SOPs) and readily accessible to the personnel. For details regarding the sequences of the probes and primers used to detect the ABL (housekeeping) gene(56) and the PML-RARA fusion gene(55), please refer to the appendix.

**Statistical Analysis**

First, the data underwent a descriptive analysis. The results were reported in the form of frequency and percentage for qualitative variables, as well as median, mean and standard deviation for quantitative variables. To assess the relationships between variables, Chi-square and Fisher exact tests were used for qualitative variables, as well as Mann-Whitney U and Independent Sample T-tests for quantitative variables.

The mean survival rate and overall survival rate of patients at different time intervals were determined using the Kaplan-Meier method. The primary comparison of mean survival rates was carried out utilizing the Log-Rank test. To identify risk factors and hazard ratios for variables, a univariate analysis was performed utilizing the proportional hazards Cox model. It was ensured that the proportional hazards assumption held for the variables included in the model.

Finally, a multivariate analysis was conducted using the multivariate Cox proportional hazards model to identify factors impacting patient survival. In addition to variables that exhibited a significant relationship with the survival rate in the univariate analysis (with a significance level of *P*<0.2), other study variables that were suggested to have a significant relationship with the survival rate in previous studies were entered into the multivariate model. Data analysis was executed using SPSS software version 26, and statistical significance was set at a level of 0.05 (two-tailed test) (59).

## RESULTS

**Demographic Features, Clinical, and Pathological Parameters**

Among the 145 newly diagnosed de novo APL patients, 75 were male, and 70 were female, with an average age of 34.26 years (2 to 78 years). Of them, 106 (73.1%) exhibited the bcr1 isoform, while 39 (26.9%) displayed the bcr3 isoform. Notably, there were no cases of the bcr2 isoform detected in our patient cohort. Consequently, the prevalence of the bcr1 isoform was significantly higher than that of the other two isoforms (*P*<0.001).

Among the patients for whom information was available:

A total of 53 patients (38.7%) had a positive family history of any type of malignancy. The most common initial symptoms were simultaneous bleeding and ecchymosis/petechia, along with other non-specific symptoms (*P*<0.001). Only 18 patients (17.6%) exhibited some degree of organomegaly. All patients had hypercellular bone marrow, with an average proportion of abnormal promyelocytes plus myeloblasts at 75.92% (ranging from 40% to 95%). Hypogranular morphology was observed in only 3 patients (2.5%). Flow cytometric immunophenotyping indicated compatibility with the diagnosis of APL in 93.8% of cases. Additionally, 23% of patients had CD34 expression. 

Conventional karyotyping of 59 patients (55.7%) revealed a t(15;17)(q22;q21) without any additional chromosomal abnormalities (i.e., 55 patients (51.9%) had t(15;17) and 4 patients (3.8%) had complex translocation between chromosomes 15 and 17 and one or two another chromosomes simultaneously). Also, 40 patients (37.7%) had a t(15;17) with additional chromosomal abnormalities, 4 patients (3.8%) only had other chromosomal abnormalities without t(15;17), and 3 patients (2.8%) had normal karyotypes. In other words, 7 patients (6.6%) had cryptic t(15;17). Overall, the most common additional chromosomal abnormality was an extra copy of chromosome 8. 

The mean WBC count was 15,000 ± 24,000 µL (400 to 130,000 µL), the platelet count was 31,000 ± 25,000 µL (7,000 to 174,000 µL), and the hemoglobin level was 8.4±2.0 mg/dL (4.1 to 14.4 mg/dL). Of the patients, 45 (36%) were classified into the high-risk group, and 80 (64%) fell into the standard-risk group. This included 60 patients (48%) in the intermediate-risk group and 20 patients (16%) in the low-risk group. Table 1 displays the patient frequency based on the variables under study, the Kaplan-Meier estimate of their mean survival time, and the results of the log-rank test for comparing the mean survival time.

**Table 1:** Demographic features and clinicopathological parameters of APL patients in the south of Iran

variables					N		%	Mean survival time(month)		P-value of Log-rank test	
Gender	Man				75		51.7	70.051		0.459	
	Woman				70		48.3	78.035			
Age	1-17y/o				17		11.7	73.261		0.067	
	18-34y/o				67		46.2	81.724			
	35-49y/o				38		26.2	73.938			
	50-85y/o				23		15.9	35.699			
Family history of cancer	Present				53		38.7	68.164			
	Absent				84		61.3	74.402		0.869	
	Unknown				8						
Onset symptom	Ecchymosis				38		27.9	81.026		0.077	
	Bleeding				21		15.4	82.983			
	Ecchymosis and bleeding				59		43.4	62.427			
	CVA or PTE or DVT				5		3.7	5.413			
	Nonspecific symptoms				13		9.6	100.346			
	Unknown				9						
Organomegaly	Present				18		17.6	73.969		0.733	
	Absent				84		82.4	73.566			
	Unknown				43						
BM morphology	Classic hypergranular				115		97.5	77.285		0.219	
	Variant hypogranular				3		2.5	N			
	Unknown				27						
BM cellularity	Hypercellular				118		100	78.583		-	
	Hypocellular				0		0	-			
	Unknown				27						
Flow cytometry	Compatible with APL				105		93.8	77.328		0.232	
	Incompatible with APL				7		6.3	84.495			
	Unknown				33						
CD34	Positive				23		23.0	52.954		0.487	
	Negative				77		77.0	79.046			
	Unknown				45						
bcr	bcr1				106		73.1	75.716		0.448	
	bcr3				39		26.9	55.329			
Additional chromosomal abnormalities	Absent		- t(15;17;*)		58		54.7	80.720		0.661	
			- t(15;17)								
			- normal karyotype								
	present		- t(15;17) with additional chromosomal abnormalities		48		45.3	84.795			
			- only other chromosomal abnormalities								
	Unknown				39						
Cytogenetic finding	**Isolated t(15;17)** (t(15;17) + t(15;17;V))				**59** (55+4)		**55.7** (51.9+3.8)	**75.040** (78.040+20.608)		**0.249** (0.236)^ *^	
	**t(15;17) with additional chromosomal abnormalities**				**40**		**37.7**	**88.384**			
	**Cryptic t(15;17)** (Only other chromosomal abnormalities + normal karyotype)				**7 **(4+3)		**6.6** (3.8+2.8)	**78.933** (69.133+N)			
	Unknown				39						
Sanz risk category	High risk				45	36.0			59.615		0.002
	Standard risk			Intermediate risk	60	48.0			73.380		
				Low risk	20	16.0			111.732		
	Unknown				20						
WBC^ **^	> 10×10^3^ /µL (High risk)				45		36.0	59.615		0.004	
	< 10×10^3^ /µL (Standard risk)				80		64.0	84.535			
	Unknown				20						
PLT	> 40×10^3^ /µL				28		22.4	88.736		0.162	
	< 40×10^3^ /µL				97		77.6	71.269			
	Unknown				20						
	> 20×10^3^ /µL				85		68.0	83.738			
PLT	< 20×10^3^ /µL				40		32.0	59.438		0.032	
	Unknown				20						
HGB	> 7 mg/dL				94		75.2	81.210		0.151	
	< 7 mg/dL				31		24.8	59.537			
	Unknown				20						
Relapse (just in CHR patients)	NO				67		79.8	121.640		<0.001	
	YES				17		20.2	78.689			
	Unknown				61						
First admission Status(all patients)		Early death			52		35.9	0.393		-	
		CHR			93		64.1	114.444			
Final status of relapsed cases	Dead				8		47.1	-		-	
	Alive				9		52.9	-			
Final status of all patients	Dead				61		42.1	-		-	
	Alive				84		57.9	-			

APL: Acute Promyelocytic leukemia, BM: Bone Marrow, CHR: Complete Hematologic Response, CVA: Cerebrovascular Attack, DVT: Deep Vein Thrombosis, HGB: Hemoglobin, PLT: Platelet, PTE: Pulmonary Thromboembolism, WBC: White Blood Cell

^* ^Statistical analysis has been done on five groups of cytogenetic findings including t(15;17), t(15;17;V), t(15;17) + additional chromosomal abnormalities, other chromosomal abnormalities, and normal karyotype

^**^ The same result with Statistical analysis on two-tier sanz hematologic risks including High risk, and Standard risk

N: Mean survival time cannot be evaluated, due to the absence of event (death) in this group.

Unknown: The patient's data is not available.

At the end of the 10-year study period, with median and mean follow-up times of 27 and 37 months, respectively (0.067 to 127.40 months), 84 patients (58%) had survived, and 61 patients (42%) had died due to the disease and its complications. Specifically, 52 (35.8%) patients had passed away during their initial hospitalization (early death), 8 patients (5.5%) had succumbed to relapse, and one patient (0.7%) had died due to septicemia caused by immune deficiency.

In fact, only 15% of all deaths occurred after the first discharge in patients who responded to induction therapy. The mean, median, and mode of the time taken from admission to early death (2 to 42 days) were 9.35, 8, and 4 days, respectively, and almost half of the early deaths occurred during the first week. Table 2 presents the frequency of patients with early death and relapse by different variables, as well as the statistical relationship between them. Only leukocytosis had a significant relationship with early death (*P*=0.002), such that the rate of early death in high-risk patients was more than twice (2.03 times) higher than in the standard-risk group (Table 2).

The mean time taken to achieve CHR was thirty-two days (15 to 64 days). Approximately one-fifth (20.23%) of patients, for whom information on relapse status was available, experienced episode(s) of relapse, and nearly half of them (47%) had passed away by the end of our study period. There was no significant relationship between relapse and any of the study variables (Table 2).

**Relationship Between PML-RARA Isoforms and Study Variables**

The bcr isoforms had a significant relationship with the number of WBCs, high-risk group (leukocytosis), cytogenetic findings, the presence of additional chromosomal abnormalities, and the time required to achieve CHR. However, no other relationship between bcr isoforms and other quantitative or qualitative variables was observed (Tables 3 and 4).

Although there was no significant difference in the rate of achieving CHR (Tables 2 and 4), on average, bcr3 patients responded faster to induction therapy than bcr1 patients (*P*=0.024). Additionally, the mean number of WBCs in bcr3 patients was significantly higher than in bcr1 patients (*P*=0.029). The proportion of high-risk cases was significantly higher in bcr3 patients than in bcr1 patients (51.4% vs. 30.0%, *P*=0.025). According to cytogenetic findings, chromosomal changes in leukemic cells of patients with the bcr3 isoform were significantly different from those with the bcr1 isoform (*P*=0.010). On the other hand, more than two-thirds of bcr3 patients (66.7%) had additional numerical and/or structural chromosomal changes, compared to less than one-third of bcr1 patients (31.6%) (*P*=0.001).

In Table 4, in addition to displaying the frequency of demographic features and disease characteristics and outcomes by different bcr isoforms, the relationship between these qualitative variables and bcr isoforms has been presented.

**Table 2:** The relationship of variables with early death and relapse rates in APL patients in South Iran

Variables			Early death			P-value	Relapse			P-value
			Yes	No			Yes	No		
bcr	bcr1		37		69	0.692^ a^	13		48	0.771^ b^
	bcr3		15		24		4		19	
Onset symptom	Ecchymosis		12		26	0.084^ b^	5		20	0.209^ b^
	Bleeding		6		15		2		12	
	Ecchymosis and bleeding		25		34		4		28	
	CVA or PTE or DVT		4		1		0		1	
	Nonspecific symptoms		2		11		5		6	
Gender	Man		28		47	0.702^ a^	11		33	0.255^ a^
	Woman		24		46		6		34	
Age	1-17y/o		6		11	0.149^ a^	1		9	0.728^b^
	18-34y/o		22		45		9		33	
	35-49y/o		11		27		6		17	
	50-85y/o		13		10		1		8	
Family history of cancer	Present		17		36	0.393^ a^	8		27	0.480^ a^
	Absent		33		51		8		40	
BM morphology	Classic hypergranular		40		75	0.550^ b^	12		58	0.999^ b^
	Variant hypogranular		0		3		0		2	
Flow cytometry	Compatible with APL		37		68	0.419^ b^	12		52	0.581^ b^
	Incompatible with APL		1		6		0		6	
CD34	Positive		9		14	0.722^ a^	2		10	0.999^ b^
	Negative		27		50		8		40	
Cytogenetic finding	Isolated t(15;17)		^23^		^36^	0.164^ b^	^3^		^29^	0.250^ b^
	t(15;17) with additional chromosomal abnormalities		9		31		7		23	
	Cryptic t(15;17)		1		6		1		4	
Additional chromosomal abnormalities	Absent		23		39	0.115^ a^	4		31	0.329^ b^
	Present		10		34		7		25	
Sanz risk category	High risk		24 19		21	0.002^ b^	3		16	0.913^ b^
	Standard risk	Intermediate risk			41		5		33	
		Low risk	2		18		3		14	
WBC*	> 10×10^3^ /µL (High risk)		24		21	0.002^ a^	3		16	0.999^ b^
	< 10×10^3^ /µL (standard risk)		21		59		8		47	
PLT	> 40×10^3^ /µL		8		20	0.353^ a^	3		16	0.999^ b^
	< 40×10^3^ /µL		37		60		8		47	
PLT	> 20×10^3^ /µL		26		59	0.066^ a^	8		47	0.999^ b^
	< 20×10^3^ /µL		19		21		3		16	
HGB	> 7 mg/dL		31		63	0.220 ^a^	8		50	0.694^ b^
	< 7 mg/dL		14		17		3		13	
Organomegaly	Present		6		12	0.775^ a^	2		10	0.999^ b^
	Absent		31		53		8		41	

APL: Acute Promyelocytic leukemia, BM: Bone Marrow, CHR: Complete Hematologic Response, CVA: Cerebrovascular Attack, DVT: Deep Vein Thrombosis, HGB: Hemoglobin, PLT: Platelet, PTE: Pulmonary Thromboembolism, WBC: White Blood Cell

^a ^Chi-squared test

^b ^Fisher exact test

* The same result with Statistical analysis on two-tier sanz hematologic risks including High risk, and Standard risk

**Table 3:** Description of quantitative study variables and their relationship with bcr isoforms

P-value		bcr 3			bcr 1			**Total**			Variables
		Mean±SD	N		Mean±SD	N		Mean±SD	N		
**0.569** ^a^		35.49±13.66	39		33.80±16.47	106		34.26±15.73	145		**Age (y/o)**
**0.024** ^b^		28.26±12.38	19		33.43±9.81	44		31.87±10.82	63		**Time to achieve CHR (day)**
**0.129** ^a^		79.16±11.79	31		74.77±14.32	87		75.92±13.79	118		**BM promyelocyte+Myeloblast(%)**
**0.029** ^a^		25.38±34.47	35		11.54±17.96	90		15.41±24.42	125		**WBC count (×10** ^3^ ** /µL)**
**0.770** ^a^		32.44±26.06	35		30.97±24.88	90		31.38±25.12	125		**PLT count (×10** ^3^ ** /µL)**
**0.322** ^a^		8.734±1.90	35		8.33±2.10	90		8.44±2.05	125		**HGB (mg/dL)**

BM: Bone Marrow, CHR: Complete Hematologic Response, HGB: Hemoglobin, PLT: Platelet, WBC: White Blood Cell

^a^ Independent-Sample T Test

^b^ Mann-Whitney Test

**Table 4:** The relationship between demographic features, disease characteristics and outcomes with bcr isoforms

Variables			bcr								P-value
			bcr1					bcr3			
			N				%	N		%	
Demographic features											
Gender	Man				55	73.3			20	26.7	0.948^ a^
	Woman				51	72.9			19	27.1	
Age	1-17y/o				14	82.4			3	17.6	0.295^ a^
	18-34y/o				49	73.1			18	26.9	
	35-49y/o				24	63.2			14	36.8	
	50-85y/o				19	82.6			4	17.4	
Family history of cancer	Present				37	69.8			16	30.2	0.611^ a^
	Absent				62	73.8			22	26.2	
Characteristics of APL disease											
Onset symptom	Ecchymosis		26				26.5	12		31.6	0.662^ b^
	Bleeding		17				17.3	4		10.5	
	Ecchymosis and bleeding		40				40.8	19		50.0	
	CVA or PTE or DVT		4				4.1	1		2.6	
	Nonspecific symptoms		11				11.2	2		5.3	
BM morphology	Classic hypergranular		86				98.9	29		93.5	0.168^ b^
	Variant hypogranular		1				1.1	2		1.7	
Flow cytometry	Compatible with APL		77				93.9	28		93.3	0.999^ b^
	Incompatible with APL		5				6.1	2		6.7	
CD34	Positive		16				21.9	7		25.9	0.672^ a^
	Negative		57				78.1	20		74.1	
Cytogenetic finding	**Isolated t(15;17)** (t(15;17) + t(15;17;*))		49 (47+2)				64.5 (61.8+2.6)	10 (8+2)		33.3 (26.7+6.7)	0.010^ a^ (0.003^ b^)^*^
	**t(15;17) with additional chromosomal abnormalities**				22		28.9	18		60.0	
	**Cryptic t(15;17)** (Only other chromosomal abnormalities + normal karyotype)		5 (2+3)				6.6 (2.6+3.9)	2 (2+0)		6.7 (6.7+0.0)	
Additional chromosomal abnormalities	Absent	- t(15;17;*)		52			68.4	10		33.3	0.001
		- t(15;17)									
		- normal karyotype									
	present	- t(15;17) with additional chromosomal abnormalities		24			31.6	20		66.7	
		- only other chromosomal abnormalities									
Sanz risk category	High risk		27				30.0	18		51.4	0.074^ a^
	Intermediate risk(Standard risk)		48				53.3	12		34.3	
	Low risk(Standard risk)		15				16.7	5		14.3	
WBC^**^	> 10×10^3^ /µL (High risk)		27				30.0	18		51.4	0.025^ a^
	< 10×10^3^ /µL(Standard risk)		63				70.0	17		48.6	
PLT	> 40×10^3^ /µL		21				23.3	7		20.0	0.688^ a^
	< 40×10^3^ /µL		69				76.7	28		80.0	
PLT	> 20×10^3^ /µL		59				65.5	26		74.3	0.347^ a^
	< 20×10^3^ /µL		31				34.5	9		25.7	
HGB	> 7 mg/dL		64				71.1	30		85.7	0.090^ a^
	< 7 mg/dL		26				28.9	5		14.3	
Organomegaly	Present		12				16.7	6		20.0	0.687^ a^
	Absent		60				83.3	24		80.0	
Outcomes											
Relapse (just in CHR patients)	No		48				78.7	19		82.6	0.771^ b^
	Yes		13				21.3	4		17.4	
First admission Status (all patients)	Early death		37				34.9	15		38.5	0.692 ^a^
	CHR		69				65.1	24		61.5	
Final status of relapsed cases	Dead		5				38.5	3		75.0	0.294 ^b^
	Alive		8				61.5	1		25.0	
Final status (all patients)	Dead		43				40.6	18		46.2	0.546 ^a^
	Alive		63				59.4	21		53.8	

APL: Acute Promyelocytic leukemia, CHR: Complete Hematologic Response, CVA: Cerebrovascular Attack, DVT: Deep Vein Thrombosis, HGB: Hemoglobin, PLT: Platelet, PTE: Pulmonary Thromboembolism, WBC: White Blood Cell

^a ^chi squared test

^b ^Fisher exact test

^* ^Statistical analysis has been done on five groups of cytogenetic findings including t(15;17), t(15;17;*), t(15;17) + additional chromosomal abnormalities, other chromosomal abnormalities, and normal karyotype

^**^ The same result with Statistical analysis on two-tier sanz hematologic risks including High risk, and Standard risk

**Survival analysis**

**Kaplan-Meier Log-Rank Test of Mean Survival Time**

**Fig. 1:**
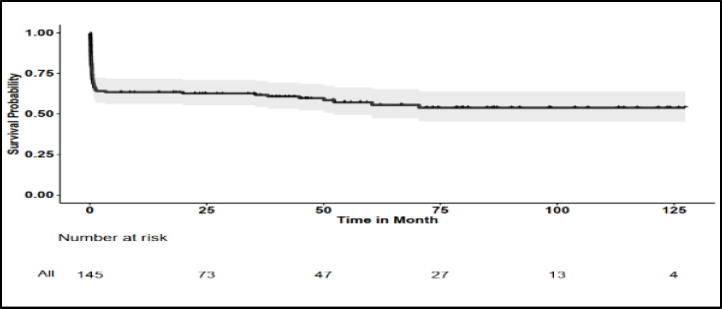
Kaplan-Meier chart of overall survival of all APL patients

**Fig. 2:**
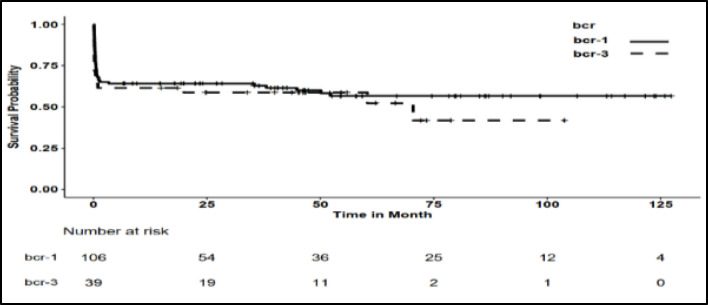
Kaplan-Meier chart of overall survival of APL patients by bcr isoforms

The Kaplan-Meier estimate of the mean survival time in patients who achieved CHR was 114.44±4.02 months. Regardless of achieving CHR, the mean survival time for for all patients, all bcr1 patients and all bcr3 patients, was 73.54±5.22 months, 75.71±6.05 months, and 55.32±8.20 months, respectively. The difference in the two bcr groups was not statistically significant (*P*=0.448) (Figures 1 and 2).

Regarding the mean survival time of patients according to study variables, only Sanz risk category, WBC, PLT, and relapse status had a significant relationship with the mean survival time (Table 1). Thus, the presence of leukocytosis (WBC > 10,000/µL) and severe thrombocytopenia (PLT < 20,000/µL) at the beginning of the disease, as well as the occurrence of relapse, were identified as risk factors that can reduce the survival of APL patients. In Table 1, the Kaplan-Meier estimate of the mean overall survival time according to the study variables, and the log-rank test to compare them, are reported. Figures 1 and 2 display the Kaplan-Meier Chart of the overall survival time for all patients and by bcr isoforms, respectively. For Kaplan-Meier Charts of other variables, please refer to the appendix.

Table 5 displays the survival probabilities at 6, 12, 24, 36, 60, 84, and 120 months for all patients, patients categorized by bcr isoforms, and those who achieved CHR. It also presents the results of comparing the survival probabilities between bcr1 and bcr3 patients at these time intervals. There was no statistically significant difference in the survival probability between the bcr1 and bcr3 groups at any of the time intervals.

**Table 5:** The survival probability of 6, 12, 24, 36, 60, 84, and 120 months in APL patients

Time (month)	Survival probability ± SD (%)				Log Rank test P-value
	All patients	CHR patients	All Patients with bcr1	All Patients with bcr3	
6	63.4 ± 4.0	98.9 ± 1.1	64.2 ± 4.7	61.5 ± 7.8	0.625
12	63.4 ± 4.0	98.9 ± 1.1	64.2 ± 4.7	61.5 ± 7.8	0.625
24	62.7 ± 4.0	97.7 ± 1.6	64.2 ± 4.7	58.7 ± 7.9	0.475
36	61.8 ± 4.1	96.3 ± 2.1	62.9 ± 4.7	58.7 ± 7.9	0.532
60	57.2 ± 4.4	89.1 ± 4.0	56.6 ± 5.2	58.7 ± 7.9	0.771
84	53.8 ± 4.7	83.9 ± 5.2	56.6 ± 5.2	41.8 ± 12.0	0.448
120	53.8 ± 4.7	83.9 ± 5.2	56.6 ± 5.2	41.8 ± 12.0	0.448

APL: Acute Promyelocytic leukemia, CHR: Complete Hematologic Response

**Univariate Cox Proportional Hazards Model**

Using the univariate Cox proportional hazards model, the relationship of various factors with the survival of APL patients was investigated (Table 6). The results of this analysis revealed that age groups, onset symptoms, relapse, WBC count, PLT count, and Sanz's risk category significantly influenced patients' survival. However, there was no significant relationship observed between the other investigated variables, such as bcr, and patients' survival.

The risk of death in patients with an age of disease onset between 18-34 years old was 56% lower than those over 50 years old (HR=0.44 (0.23-0.83)) (*P*=0.012). Patients who presented with thrombotic events (e.g., CVA, PTE, or DVT) at the beginning of the disease had a 5.3 times higher risk of death compared to patients who presented only with other non-specific symptoms (HR=5.32 (1.18-23.96)) (*P*=0.029). Patients who experienced even one episode of relapse had a 29 times higher risk of death than those who did not (HR=28.78 (3.6-230.33)) (*P*=0.002). Patients with primary leukocytosis (high-risk group) had a 2.2 times higher risk of death than those in the standard risk group (HR=2.20 (1.26-3.84)) (*P*=0.006). Additionally, concerning the quantitative measurement of WBCs, for every 1000 cells/µL increase in the WBC count, the risk of death increased by 2% (HR=1.02 (1.01-1.03)) (*P*=0.006). Regarding the initial PLT count, patients with platelets < 20,000/µL had an 81% higher risk of death compared to others (HR=1.81 (1.03-3.17)) (*P*=0.038). Furthermore, for every 1000 cells/µL increase in the platelet count, the risk of death decreased by 2% (HR=0.98 (0.96-0.99)) (*P*=0.014).

**Table 6:** Factors associated with survival of APL patients (univariate Cox proportional hazards model)

Variables		HR (95% CI)	P-value
bcr isoforms	bcr1/ bcr3	0.81 (0.47-1.4)	0.453
Gender	Man/Woman	1.21 (0.73-2)	0.464
Age group	50-85 y/o		
	1-17 y/o	0.52 (0.21-1.28)	0.155
	18-34 y/o	0.44 (0.23-0.83)	0.012
	35-49 y/o	0.50 (0.24-1.02)	0.058
Family history of cancer	Present/Absent	1.05 (0.62-1.77)	0.870
Onset symptom	Nonspecific symptoms		
	Ecchymosis	1.87 (0.54-6.52)	0.324
	Ecchymosis and bleeding	1.68 (0.43-6.51)	0.451
	Bleeding	2.95 (0.89-9.68)	0.075
	CVA or PTE or DVT	5.32 (1.18-23.96)	0.029
Flow cytometry	Compatible with APL /Incompatible with APL	3.12 (0.43-22.72)	0.260
CD34	Positive /Negative	1.28 (0.64-2.56)	0.492
Cytogenetic finding	**Isolated t(15;17)** (t(15;17) + t(15;17;*))		
	**t(15;17) with additional chromosomal abnormalities**	0.67 (0.34-1.34)	0.257
	**Cryptic t(15;17)** (Only other chromosomal abnormalities + normal karyotype)	0.28 (0.04-2.06)	0.211
Additional chromosomal abnormalities	Present/Absent	0.70 (0.35-1.37)	0.292
Sanz risk category	High risk / Standard risk	2.20 (1.26-3.84)	0.006
WBC count		1.02 (1.01-1.03)	0.006
WBC	>10×10^3^/µL / < 10×10^3^ /µL	2.20 (1.26-3.84)	0.006
PLT count		0.98 (0.96-0.99)	0.014
PLT	< 40×10^3^ /µL / > 40×10^3^ /µL	1.70 (0.80-3.63)	0.170
PLT	< 20×10^3^ /µL / > 20×10^3^ /µL	1.81 (1.03-3.17)	0.039
HGB mg/dl		0.86 (0.74-0.99)	0.055
HGB	< 7 mg/dL / >7 mg/dl	1.54 (.85-2.79)	0.158
Time to CHR		1.01 (.90-1.14)	0.834
Organomegaly	Present/Absent	0.87 (0.39-1.96)	0.735
Relapse(just in CHR patients)	Yes/No	28.78 (3.6-230.33)	0.002

APL: Acute Promyelocytic leukemia, BM: Bone Marrow, CHR: Complete Hematologic Response, CVA: Cerebrovascular Attack, DVT: Deep Vein Thrombosis, HGB: Hemoglobin, PLT: Platelet, PTE: Pulmonary Thromboembolism, WBC: White Blood Cell

**Multivariate Cox Proportional Hazards Model**

Using the multivariate cox proportional hazards model, factors related to the survival of APL patients were investigated. Among the variables previously examined in the univariate model, we included bcr isoform, gender, age group, family history of malignancies, onset symptom, additional chromosomal abnormalities, WBC count, PLT count, and HGB level in the multivariate Cox model (Table 7).

**Table 7:** Factors associated with survival of APL patients (multivariate Cox proportional hazards model)

Variables		HR (95% CI)	P-value
bcr	bcr1/ bcr3	0.75 (0.3-1.84)	0.526
Gender	Man/Woman	1.08 (0.51-2.28)	0.843
Age group	50-85		0.088
	1-17	0.45 (0.11-1.79)	0.255
	18-34	0.28 (0.1-0.75)	0.011
	35-49	0.53 (0.19-1.53)	0.241
Family history of cancer	Present/Absent	0.94 (0.43-2.07)	0.880
Onset symptom	Nonspecific symptoms		0.433
	Ecchymosis	0.81 (0.15-4.43)	0.809
	Ecchymosis and bleeding	0.96 (0.15-6.33)	0.969
	Bleeding	1.72 (0.32-9.28)	0.526
	CVA or PTE or DVT	2.59 (0.31-21.49)	0.378
Additional chromosomal abnormalities	Present/Absent	1.88 (0.78-4.51)	0.158
WBC count/µL		1.03 (1.01-1.04)	0.002
PLT count/µL		0.97 (0.94-1)	0.076
HGB mg/dl		0.86 (0.69-1.06)	0.161

APL: Acute Promyelocytic leukemia, CVA: Cerebrovascular Attack, DVT: Deep Vein Thrombosis, HGB: Hemoglobin, PLT: Platelet, PTE: Pulmonary Thromboembolism, WBC: White Blood Cell

After controlling for the effects of other factors, only the variables of age groups and WBC count still maintained their relationship with the survival of APL patients. Thus, the risk of death in patients who were between 18-34 years old was 72% lower than in patients over 50 years old (*P*=0.011), and for every 1000 cells/µL increase in WBC count, the risk of death increased by 3% (*P*=0.002).

## Discussion

**Epidemiological Investigation of APL Patients in Iran**

In our study, the prevalence of bcr isoforms in APL patients was as follows: 73% for bcr1, 27% for bcr3, and 0% for bcr2. These findings align with prior research, consistently reporting a higher prevalence of bcr1 isoforms compared to bcr3 (9, 13-19, 33, 35, 36, 40, 42, 44, 60-64). Additionally, the prevalence of the bcr2 isoform was exceedingly low, consistent with previous studies (20, 33, 38-40, 44, 61).

It's worth noting that some Indian and Pakistani researchers have reported the S isoform as the dominant one in their studies. They attributed these differences in isoform prevalence to genetic factors and environmental influences unique to different populations (20, 21, 34, 43). 

In our study, which was conducted in a referral center located in the south of Iran with both pediatric and adult departments, we included all patients without an age limit who tested positive for the PML-RARA gene through PCR testing. Our observations revealed that the mean age of disease onset was 34.26 years old, with the largest proportion of patients (46.2%) falling into the 18 to 34 years old age group. These findings are consistent with the results of a few previous studies conducted on the Iranian population, as well as some other studies, which reported a mean age of disease onset between 31 and 35 years old (21, 34, 36, 61, 65, 66).

While in scientific sources, the mean age of APL disease in the world is reported to be around 40 y/o (between 30 and 40 y/o) (48, 67, 68). In two large studies conducted on a large number of patients with APL during 1975 to 2010 using the National Cancer Institute SEER registry data in the United States, the mean age of disease onset was reported to be 44 y/o (69) and 48 y/o (70). On the other hand, there are many other studies, especially on Asian, Latin and South American communities, which have reported the mean age of onset of APL in the third decade (9, 20, 38, 40, 62). These differences can be considered as a result of differences in the age distribution of societies or due to the smaller sample size in some studies. What can be concluded from our study, which is consistent with other studies, is that the most common age of APL is middle-aged and is lower than other AMLs (64). 

Another finding of this study was the same prevalence of the disease in both men and women, which was consistent with the findings of other studies and reference scientific sources in this field (9, 34, 37, 48, 63, 64, 70, 71). Although many other studies have reported that more men were affected (1, 17, 18, 36, 43, 65, 72-75) and in a smaller number, more women were affected (20, 39, 61, 63, 76, 77), the existing difference was often small and these studies mostly had a relatively small sample size. On the other hand, the differences in gender and age distribution between the studied communities can be another cause of these different reports.

Where almost one-third of the patients in this study had a positive family history of various malignancies, this has not been found in any other previous literature. In order to better understanding the impact of genetic predisposition to cancer, as a prognostic or predictive factor, more research should be done in the future.

In this study, it was observed that more than 90% of patients were referred to medical centers with at least one of the symptoms caused by hemorrhagic and coagulopathic disorders related to DIC as the main complaint, and only about 10% were referred due to the presence of non-specific symptoms. Nearly half of the patients (43.4%) had reported the simultaneous presence of ecchymotic lesions and bleeding at the beginning of their disease, and less than 4% of them were diagnosed with thromboembolic disorders (e.g., CVA, PTE, and DVT). These results are completely consistent with the current scientific references and clearly show the importance of APL disease as a hematological emergency (72, 75, 78). 

As mentioned in textbooks, organomegaly (hepatomegaly and/or splenomegaly) is not a common symptom in APL(78) where only one sixth of our patients (17.6%), had a mild degree of organomegaly, which was lower than some previous studies (66, 72) and higher than some others (34). 

All patients had hypercellular bone marrow in accordance with the scientific references(48) where the mean percentage of myeloblast/promyelocyte in their bone marrow was 75.92%, which was in line with the findings of other researchers(36, 65). Of patients, 97.5% had hypergranular promyelocyte (classic variant) with only 2.5% having the hypogranular variant (hypogranular/microgranular subtype, M3V). This is consistent with the results of some studies in India, Egypt, and Iran (20, 21, 40, 61, 65). Since in textbooks and many past studies, the hypogranular variant has been reported in 15-25% of all APL cases (1, 13, 17, 34, 39, 42, 48). The reason for this difference can be attributed to the lack of proper identification of these patients as APL (due to morphological differences) or the real difference in its prevalence in different populations or even the difference in the sample size of the investigated patients in different studies.

Of patients, 93.7% had immunophenotyping findings compatible with APL diagnosis (48), which confirms the value of flow cytometry in the early diagnosis of this disease. Also, 23% of patients expressed CD34 on their malignant cells, which was consistent with the findings of previous studies that reported CD34 expression to be around 20-30% (39, 79-82). However, in some studies, a lower level of CD34 expression (7 to 10%) has been reported (38, 65).

Of patients, 66% despite confirmed presence of PML-RARA gene through PCR (as inclusion criteria), had undetectable t(15;17) in the conventional karyotype of their malignant bone marrow cells, which is similar to reports in some past studies(44, 62). Also, 45.6% of all patients had other additional numerical and/or structural chromosomal abnormalities, the most common of which was +8, which was in good agreement with the scientific sources (48), but in some studies, the presence of additional chromosomal abnormalities was reported in a smaller number of patients (about 20 to 30%) (44, 62, 63, 79, 83). An Indian study observed additional chromosomal abnormalities in only 7% of APL patients(38). 

As reported in other studies and scientific sources regarding patients with APL, in the present study the majority of APL patients had leukocytopenia and almost all of them had thrombocytopenia and anemia (18, 48, 84). According to Sanz's risk grouping, more than one third (36%) of our patients were in the high-risk group and the other 64% were in the Standard-risk group (including 48% in the Intermediate-risk group and 16% in the Low-risk group). This is completely consistent with the findings of some previous researchers (62, 76). Although in some studies, a different prevalence rate has been reported for the high-risk group (as an example this rate was 85% (65) in an Iranian study), in many reliable sources and other studies, the prevalence of the high-risk group in APL is limited to 10-30% of patients (18, 36, 42, 48, 84, 85).

Of all our patients, 36% died in the first hospitalization. This means some of the patients had died before the start of treatment within the first few days of hospitalization and some had died despite the initiation of induction therapy for them (in this study, detailed information on this issue was not available for some patients). The median and mode of the duration from admission to early death were 8 and 4 days respectively. That is similar to the study of Javier de la Serna and his colleagues where half of early deaths occurred in the first week (59). Also, our results were consistent with the results of other researchers that 29% of all patients suffer early death, and about a third of them die before starting treatment (86). Breccia et al also reported an early death rate of 5 to 10% in developed countries and 20 to 30% in less developed countries and considered the cause of early death to be the lack of early diagnosis, late start of treatment, and a higher WBC count (87). In this study, consistent with the results of some other researchers, early death had a significant relationship with WBC, and the early death rate in the high-risk group was more than twice (2.03) higher than in the Standard risk group (59, 87). McClellan* et al. *reported a 20% early death rate in patients who had been initiated on induction therapy (76), and it seems that despite significant advances in the treatment of APL patients (as the most curable type of leukemia), the relatively high rate of early death remains as one of the worrying reasons regarding this disease. 64% of all our patients achieved a hematological response after receiving the standard induction treatment with a mean of 31.87 days. In an Indian study in 2007, where patients were treated with ATO single-drug regimen, the mean duration was reported to be 43.7 days (63). Although there are very few studies in this regard and we did not find more data in this field, it seems that this difference could be caused by the difference in the treatment regimen used for patients. Therefore, it is suggested to conduct more studies in the future.

In two studies by Rostami* et al. *(61) and Asvadi* et al. *(65) that were conducted in Iran, the CHR rate was reported as 96.5% and 57.1% respectively. In many other studies, this rate was reported as 70 to 80% (20, 21, 34, 36, 38, 62, 88) and some have mentioned the CHR rate as 90% (59, 63, 89). This variety in CHR rate in studies conducted around the world can be because of the differences in the number of observed patients, the speed of diagnosis and initiation of treatment, the type of treatment regimen and available drugs in different time periods. On the other hand, the higher CHR rate in some studies is due to the fact that patients who died before the start of treatment were not included in the calculation (89). 

About 20% of our patients who achieved CHR had episodes of relapse. In a review study, Joaquin J. Jimenez reported the relapse rate of APL patients, regardless of their Sanz risk category, about 10 to 20% (5). By the end of our study period, more than half (53%) of the patients who had relapsed survived, and this observation confirmed the findings of Martin S. Tallman* et al. *(85). In line with the result of Seuza Melo* et al. *(62), about 10% of our patients who had reached CHR died by the end of this 10-years study period.

At the end of our study period, with a median and mean follow-up period of 27 and 37 months respectively, 42% of all patients had died due to the disease and complications caused by the treatment. 85% of deceased patients had early death at the beginning of their disease diagnosis, especially in the first week, and only 15% of all deaths occurred in patients who had reached CHR. This finding clearly highlights the importance of early and correct management of this hemato-oncology emergency.

**Relationship Between PML-RARA Isoforms and Study Variables**

Regarding the potential relationship between PML-RARA isoforms and various studied variables in Iranian APL patients, our results unveiled a significant association between bcr3 isoform and specific factors such as leukocytosis (high-risk group) and higher WBC count, a finding well-documented in numerous studies and reference texts (17, 18, 30, 35, 38). However, it's important to acknowledge that there are many other studies that have not identified any significant relationship between bcr isoforms and Sanz risk category (13, 20, 36), or WBC count (1, 9, 13, 20, 21, 40, 44). 

In terms of the presence of additional chromosomal abnormalities in leukemic cells, our bcr3 patients exhibited more than twice the prevalence compared to bcr1 patients. However, previous studies, conducted on significantly smaller cohorts of patients, did not establish this relationship (18, 35, 38, 62). Additionally, we found that bcr3 patients responded to standard induction treatments in a significantly shorter time frame than bcr1 patients. To the best of our knowledge, no previous study has explored the relationship between bcr isoforms and the time required to achieve CHR.

In our study, consistent with numerous other research findings, no statistically significant differences were observed between two patient groups categorized by bcr1 and bcr3 isoforms in terms of various factors, including gender (1, 9, 13, 17, 18, 20, 21, 30, 35, 36, 38, 43), age (1, 9, 13, 17, 18, 20, 21, 30, 35, 36, 38, 43) , family history of malignancies, bone marrow morphology (9, 13, 35, 38, 44), percentage of bone marrow myeloblasts and promyelocytes (36, 38), platelet count (1, 9, 18, 20, 30, 35, 38, 40), hemoglobin levels (1, 9, 13, 18, 30, 38, 40), onset symptoms (1, 17, 18, 35, 40), organomegaly (18), flow cytometry findings (38), CD34 expression (38), rate of early death or achievement of CHR (1, 17, 18, 20, 33, 35, 38, 39, 60, 87), relapse rate (36, 44), mortality rate in relapsed cases, overall mortality rate in all patients, and mean overall survival (18, 35). However, some studies have reported that the bcr3 isoform is associated with a lower age of disease onset (37), M3v morphology (17, 18, 30), higher percentages of bone marrow myeloblasts/promyelocytes (17), lower platelet counts(36), CD34 expression(18, 37, 39, 80, 81), higher rates of early death or lower rates of achieving CHR (36, 40), a higher relapse rate(33, 35, 36), and poorer overall survival (18, 30-34, 36).

In most available scientific reports, comparisons have been made primarily between the S and L isoforms, and it seems that due to the very low prevalence of the V isoform, its relationships with various factors could not be statistically investigated. However, there are a few studies that did not find any significant differences between the three isoforms (V, L, and S) concerning these significant relationships (1, 9, 13, 20, 21, 43, 44), possibly due to the small number of patients examined in these studies (1). Although the clinical and prognostic implications of PML-RARA isoform differences in APL patients have not yet been clearly defined (23, 29), the existing studies suggest the importance of determining PML-RARA isoform type in the early stages of disease diagnosis.

**Survival Analysis**

In the next phase of our study, we delved into the relationship between various study variables and patient survival outcomes. Over the course of the 10-year study period, with a median follow-up duration of 27 months, 58% of all patients managed to survive. The 120-month probability of survival (survival rate) was determined to be 83.9% for patients who achieved CHR and 53.8% for all patients combined. These findings align with some prior studies that reported a 10-year survival rate of 77%(90). For instance, in a 2014 study by Shetty* et al. *focusing on the survival of patients who reached CHR, the probabilities of survival at 6.3 years and 7.9 years were reported as 88% and 82%, respectively(88).

In our study, the mean overall survival time was 73.5 months. In accordance with certain earlier investigations, several variables were identified as risk factors for overall survival, including age groups(48, 66), risk group classification(34, 66), WBC count(38, 39, 62), PLT count(66), onset symptoms, and relapse. While some studies, such as those conducted by Nath and Melo, did not find a significant relationship between the age of disease onset and overall survival(38, 62), and Joseph G. Jurcic failed to establish a connection between overall survival and WBC count(33), our study, after adjusting for the effects of other factors using the multivariate Cox proportional hazards model, confirmed that age(40) and WBC count(34) maintained their association with patient survival.

On the contrary, in line with certain studies, we did not find any significant relationship between patient survival and several other variables, including bcr isoform (21, 30, 33, 38, 39, 44), gender (38), flow cytometry findings (38), CD34 expression (38, 39), cytogenetic findings and the presence of additional chromosomal abnormalities (48, 63, 79, 83, 89), organomegaly, family history of malignancies, and hemoglobin levels. However, it's important to note that some researchers in their studies have identified bcr3 isoform (34, 36), male gender (48, 66, 80, 90), and CD34 expression (80) as unfavorable prognostic factors.

## Conclusion

In conclusion, despite the numerous studies conducted on APL patients worldwide, the results highlight the lack of consensus on potential prognostic factors and the possible association between bcr isoforms and various prognostic and demographic variables. These discrepancies can be attributed to several limitations, including small sample sizes in most studies, challenges in collecting accurate data in large retrospective studies, variations in diagnostic and treatment resources across different regions, genetic diversity, and variations in the prevalence of certain intervening mutations in different populations. To enhance the management of APL patients, it is imperative to conduct large prospective cohort studies with strict data control, encompassing diverse APL patient populations in various geographical regions across the globe.

## Funding and Sponsorship

This research received no specific grant from any funding agency in the public, commercial, or not-for-profit sectors.

## Conflict of Interest

There are no conflicting interests.
